# The Protective Effects of Melatonin on Aluminum-Induced Hepatotoxicity and Nephrotoxicity in Rats

**DOI:** 10.1155/2020/7375136

**Published:** 2020-10-19

**Authors:** Mohamed S. Othman, Mohamed A. Fareid, Reda S. Abdel Hameed, Ahmed E. Abdel Moneim

**Affiliations:** ^1^Basic Sciences Department, Preparatory Year, University of Ha'il, Ha'il, Saudi Arabia; ^2^Chemistry Dept., Faculty of Biotechnology, October University for Modern Science and Arts (MSA), Giza, Egypt; ^3^Botany and Microbiology Department, Faculty of Science, Al-Azhar University, Cairo, Egypt; ^4^Chemistry Department, Faculty of Science, Al-Azhar University, Cairo, Egypt; ^5^Department of Zoology and Entomology, Faculty of Science, Helwan University, Cairo 11795, Egypt

## Abstract

Aluminum (Al) is a ubiquitous element with known toxicity for both humans and animals. Herein, we aimed to investigate the potential role of melatonin (MEL) in hepatotoxicity and nephrotoxicity following aluminum chloride (AlCl_3_) treatment in rats. Adult male rats were treated with AlCl_3_ (34 mg/kg bwt) for eight weeks. Exposure to AlCl_3_ enhanced the serum activities of the liver transaminases (alanine aminotransferase and aspartate aminotransferase) and increased the level of bilirubin, in addition to the serum kidney function markers urea and creatinine. AlCl_3_ intoxication boosted oxidative stress, as evidenced by increases in the levels of lipid peroxidation (LPO) and nitric oxide (NO) along with simultaneous decreases in the levels of glutathione (GSH), various antioxidant enzymes, and *Nrf2* mRNA expression. MEL (5 mg/kg bwt) treatment repressed LPO and NO levels, whereas it augmented GSH content. The activities of the antioxidant enzymes GPx, SOD, CAT, and GR were also restored concomitantly when MEL was administered before AlCl_3_. MEL also suppressed the apoptotic effect of AlCl_3_ by enhancing Bcl-2 protein expression in the liver and kidney and decreasing the expression levels of proinflammatory cytokines. Histopathological findings in the liver and kidney tissues confirmed the beneficial effect of MEL against AlCl_3_ toxicity. These findings indicate that MEL prevents AlCl_3_ toxicity by enhancing the antioxidant defense system.

## 1. Introduction

Humans and animals interact daily with their environment and are exposed to a wide range of chemicals and heavy metals, which can bioaccumulate in the body and collect in tissues with low excretion [1]. The third most abundant metallic element in Earth's crust is aluminum (Al) [2]. Avoiding exposure to Al is almost impossible as it is used in various daily applications, such as water treatments, wood preservation, shampoos, vitamins, food additives, packaging materials, antiperspirants, toothpaste, medicines, or as fillers in plastics [3]. Commonly, Al reaches humans by breathing ambient air and intaking contaminated food and water [4]. Furthermore, particulate matters produced by cement factories and industrial wastewater contain a high amount of Al, which leads to exposure to higher than the allowable levels of Al [5]. The average weekly intake of Al is estimated to be nearly 70–140 mg, and even with low gastrointestinal absorption capacity (less than 1%), Al might concentrate over time in essential organs, such as the brain, liver, and kidney, with apparent neurotoxicity and cytotoxicity. Hence, Al is included in the priority list of dangerous materials authorized by the Agency for Toxic Substances and Disease Registry (ATSDR) [3].

Several reports have demonstrated that the in vivo and in vitro toxicities of Al negatively affect cellular structure and macromolecules, which lead to cytotoxicity, ROS generation, mitochondrial dysfunction, inflammation, cell death, genetic damage, and carcinogenicity [5]. Sun et al. [6] reported that excessive exposure to Al exerts toxic effects on the nervous, respiratory, reproductive, and immune systems, in addition to the liver and bone. Meanwhile, Lentini et al. [7] reported that much of the Al that accumulates in the human body comes from contaminated food and water, and a smaller amount enters through the skin. The majority of this Al is rapidly removed by the kidneys, which leads to nephrotoxicity and renal impairment. Additionally, Wang et al. [8] concluded that Al accumulation in the hepatic tissue causes hepatotoxicity, and Al-Kahtani et al. [9] recently found that Al-induced hepatotoxicity causes oxidative stress and apoptosis in rats.

Melatonin (MEL) is synthesized and secreted by the pineal gland. It regulates many biological functions, such as the circadian rhythm, sleep, reproduction, and immunity [10]. Many studies have indicated that MEL has several pharmacological effects, including antioxidant, anticancer, anti-inflammatory, antiapoptotic, and immunomodulatory effects [10, 11]. In particular, Al-Olayan et al. [2] concluded that MEL is a pleiotropic hormone that exerts efficient protection against oxidative/nitrosative injury by various mechanisms.

Since there is always a need for an outstanding therapeutic agent that could suppress the initiation and progression of hepatotoxicity and nephrotoxicity, the current investigation was designed to determine the potential effect of exogenous MEL in modulating Al-induced toxicity and oxidative stress as well as its effect on nuclear factor erythroid 2-related factor 2 (Nrf2) gene expression in the liver and kidney tissues.

## 2. Materials and Methods

### 2.1. Chemicals

All the chemicals used in this study were of analytical grade and were purchased from Sigma-Aldrich Chemical Co. (St. Louis, MO, USA). Aluminum chloride (AlCl_3_) anhydrous (CAS number 7446-70-0) was dissolved in 0.9% sodium chloride solution, whereas MEL (CAS number 73-31-4) was dissolved immediately before its use in a mixture of 1% pure ethanol (96%) and 99% saline.

### 2.2. Animals

Ten-week-old male Sprague Dawley rats (200–220 g) were placed in suitable stainless steel cages (5 rats/cage). The animals were reared under a controlled lab environment (dark–light cycle of 12 hours, temperature of 22 ± 3°C, and relative humidity of 50 ± 10%), and food and tap water were provided *ad libitum*. After one week of adaptation, the rats were randomly selected and allocated into four groups (*n* = 7); the control (CON) group was administered physiological saline containing 1% ethanol, the AlCl_3_ group received 34 mg of AlCl_3_/kg bwt (1/25 LD_50_) in accordance with the study by Yousef and Salama [12], the MEL group was injected 5 mg of MEL/kg bwt, and the MEL+AlCl_3_ group was injected with 5 mg of MEL/kg bwt for 30 minutes before the administration of AlCl_3_. Doses of MEL and AlCl_3_ were administered once daily for eight weeks.

This study was reviewed and approved by the Ethical Committee of the University of Ha'il (number 1 on 25/8/1440H). It was also conducted in accordance with the European Community Directive (86/609/EEC) and the national rules on animal care, which follow the NIH Guidelines for the Care and Use of Laboratory Animals (8th edition).

### 2.3. Sample Collection

Twenty-four hours after the last administration, the rats were sacrificed under anesthesia, and then their blood was collected and centrifuged to obtain serum for biochemical assays. The liver and kidney were quickly collected, weighed, cleaned, and then cut into small pieces (100 mg each). One of the pieces from each group was homogenized in cold phosphate buffer (pH 7.4) and then centrifuged at 4000 × *g* for 10 minutes at 4°C. The resulting supernatants were used for biochemical analyses. The remaining pieces of the animals' liver and kidney were frozen at −80°C until they were used for Al determination and gene and protein expression analyses.

### 2.4. Biochemical Analyses

#### 2.4.1. Al Levels in the Animals' Liver and Kidney Tissues

The Al levels in the hepatic and renal tissues were determined by graphite furnace atomic absorption spectrophotometry (GFAAS; Perkin-Elmer 3100) in accordance with a previous study [13]. Al levels are presented as micrograms per gram of wet tissue.

#### 2.4.2. Liver and Kidney Function Tests

For the hepatic and renal function tests, the levels of alanine aminotransferases (ALT), aspartate aminotransferases (AST), bilirubin, creatinine, and urea in serum were determined using specific commercial kits from BioSystems S.A. (Barcelona, Spain) according to the manufacturer's instructions.

#### 2.4.3. Markers of Oxidative Stress

Reduced glutathione (GSH), lipid peroxidation (LPO), and nitric oxide (NO) levels in liver and kidney homogenates were determined using the methods of Giustarini et al. [14], Schaffazick et al. [15], and Bryan and Grisham [16], respectively.

#### 2.4.4. Activities of Antioxidant Enzymes

The activities of superoxide dismutase (SOD) and catalase (CAT) in hepatic and renal homogenates were determined by the methods described by Sun et al. [17] and Luck [18], respectively. The activities of glutathione reductase (GR) and glutathione peroxidase (GPx) were analyzed by the procedures developed by Factor et al. [19] and Weydert and Cullen [20], respectively.

#### 2.4.5. *Nrf2* Gene Expression Level

The expression levels of the *Nrf2* gene in hepatic and renal tissues were determined by real-time PCR using an ABI PRISM 7500 machine with SYBR Green PCR Core Reagents (Applied Biosystems, Waltham, MA, USA). The PCR amplification conditions were performed for 10 minutes at 95°C, followed by 40 cycles of 15 s at 95°C, and 1 minute at 60°C. The *β*-actin gene was used as a housekeeping gene for normalization. The results are expressed relative to those of the control group, which were normalized to 1. The primer pairs used in this process are shown in Table 1.

### 2.5. Western Blot Analysis

The nuclear protein extraction and western blot analysis were performed following the methods described by Almeer et al. [21]. The utilized antibodies included mouse anti-Nrf2 (sc-28379, 1 : 500; Santa Cruz Biotechnology, Santa Cruz, CA, USA), mouse antihistone H1 (sc-393358, 1 : 1500; Santa Cruz Biotechnology), and goat antimouse IgG (sc-2039, 1 : 5,000; Santa Cruz Biotechnology). Immunoblot analyses of antimouse IgG horseradish peroxidase-conjugated antibodies were performed using an enhanced chemiluminescence detection kit (Bio-Rad, USA). Data were acquired in arbitrary densitometric units using the ImageJ software.

### 2.6. Assessment of Tissue Proinflammatory Markers

The levels of tumor necrosis factor-alpha (TNF-*α*; Cat # CSB-E11987r) and interleukin-1 beta (IL-1*β*; Cat # CSB-E08055r) in the tissues were measured using rat ELISA kits (CUSABIO Life Sciences, China) according to the manufacturer's instructions.

### 2.7. Histopathological Studies

Small parts of the hepatic and renal tissues were fixed in 10% paraformaldehyde, embedded in paraffin blocks, sectioned (4–5 *μ*m), and then stained with hematoxylin and eosin for microscopic examination.

### 2.8. Immunohistochemical Analysis

The immune localization of Bcl-2 was analyzed using the method described by Pedrycz and Czerny [22]. Photographs were captured at 400x magnification using an Eclipse E200-LED microscope (Nikon, Tokyo, Japan).

### 2.9. Statistical Analyses

Data were expressed as mean ± standard deviation (SD). One-way analysis of variance (ANOVA) followed by Tukey's post hoc test was applied using SPSS (IBM SPSS Statistics for Windows, Version 20.0, 2011, Armonk, NY) to calculate the differences between groups; *p* values of <0.05 were considered significant.

## 3. Results

### 3.1. Aluminum Levels in Hepatic and Renal Tissues

**Figure 1:**
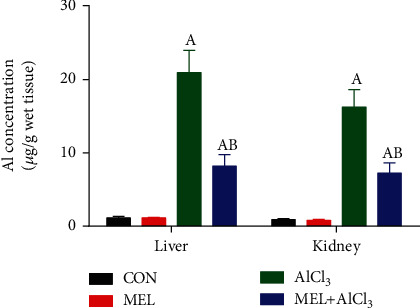
Protective effects of melatonin on the aluminum accumulation in liver and kidney tissues of rats exposed to aluminum chloride (AlCl_3_). Data are expressed as mean ± SD values (*n* = 7). ^a^*p* < 0.05 vs. the control group; ^b^*p* < 0.05 vs. the AlCl_3_-intoxicated group, using Tukey's post hoc test.

As shown in Figure 1, there was a marked (*p* < 0.05) increase in the Al levels in the hepatic and renal tissues of rats in the AlCl_3_ group compared with those in the CON group. However, the administration of MEL prior to AlCl_3_ extensively reduced (*p* < 0.05) the levels of Al in the hepatic and renal tissues of rats in the MEL+AlCl_3_ group compared with those in the AlCl_3_ group. No significant changes were observed in the levels of Al in the hepatic and renal tissues among rats in the MEL group.

### 3.2. Results of Liver and Kidney Function Tests

To explore the effect of MEL on AlCl_3_-induced hepatic and renal dysfunctions, liver and kidney function markers in the serum of rats were analyzed after the administration of AlCl_3_ for eight weeks. As shown in Table 2, there were significant (*p* < 0.05) increases in the level of bilirubin and the activities of ALT and AST in the AlCl_3_ group compared with those in the CON group. Similarly, the serum levels of creatinine and urea were markedly (*p* < 0.01) higher in the AlCl_3_ group than in the CON group. Pretreatment with MEL significantly (*p* < 0.05) prevented the disturbance in the liver and kidney markers.

### 3.3. Results of Oxidative Stress Markers and Antioxidant Enzyme Activities

**Figure 2:**
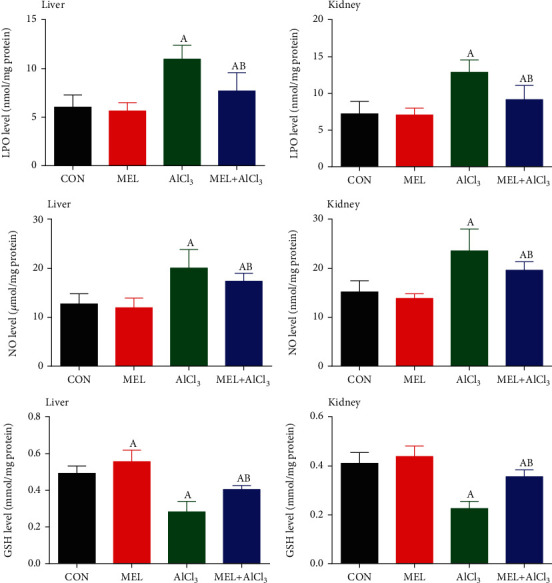
Protective effects of melatonin on aluminum chloride (AlCl_3_-) induced oxidative stress markers (LPO, NO, and GSH) in the liver and kidney tissues of rats. Data are expressed as mean ± SD values (*n* = 7). ^a^*p* < 0.05 vs. the control group; ^b^*p* < 0.05 vs. the AlCl_3_-intoxicated group, using Tukey's post hoc test.

**Figure 3:**
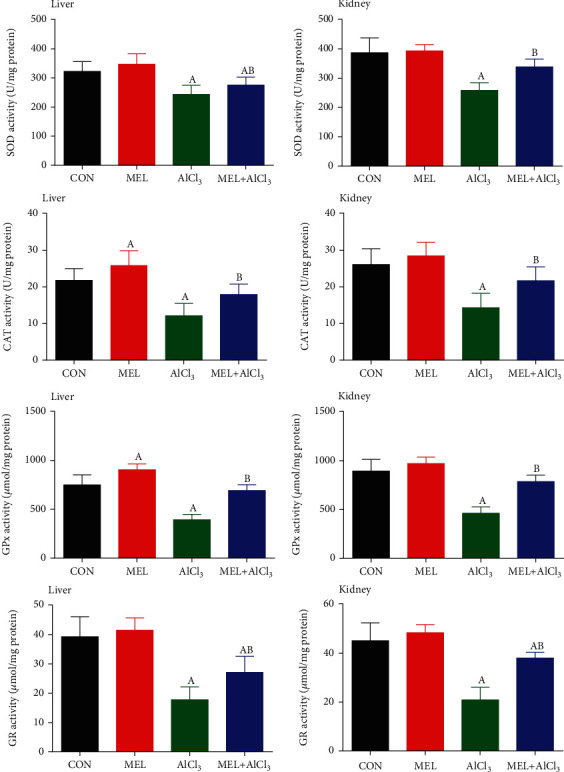
Protective effects of melatonin on aluminum chloride (AlCl_3_-) induced inhibition in the antioxidant enzyme activities (SOD, CAT, GPx, and GR) in the liver and kidney tissues of rats. Data are expressed as mean ± SD values (*n* = 7). ^a^*p* < 0.05 vs. the control group; ^b^*p* < 0.05 vs. the AlCl_3_-intoxicated group, using Tukey's post hoc test.

The manifested AlCl_3_-induced oxidative stress in hepatic and renal tissues was caused by significant (*p* < 0.05) increases in the levels of LPO and NO concurrently with a significant (*p* < 0.05) decrease in GSH contents and inhibition of antioxidant enzyme activities in comparison with the CON group. Pretreatment with MEL alleviated these alterations in LPO and GSH levels and antioxidant enzyme activities as shown in Figures 2 and 3. Moreover, the administration of MEL alone improved the GSH content and antioxidant enzyme activities of the hepatic and renal tissues when compared with the CON group.

### 3.4. *Nrf2* mRNA Expression

**Figure 4:**
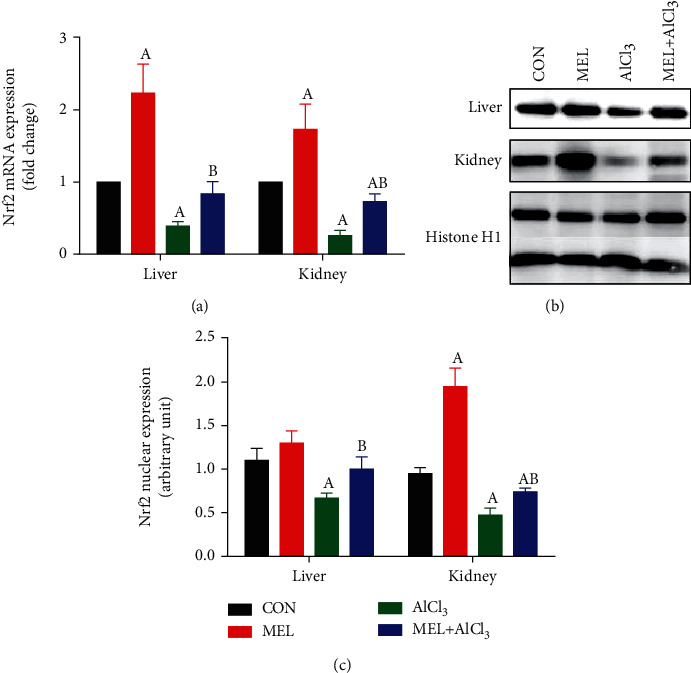
Protective effects of melatonin on aluminum chloride (AlCl_3_-) induced downregulation in (a) *Nrf2* mRNA expression in the liver and kidney tissues of rats and (b) western blot analysis of Nrf2 protein in the liver and kidney tissues of rats, and (c) quantification of the density of expression level of Nrf2. mRNA data were normalized with *β*-actin and represented as fold change (log2 scale) as compared to mRNA levels from the control rats, and western blot data were normalized with histone H1 and represented as fold change as compared to protein levels from the control rats. Data are expressed as mean ± SD values (*n* = 5). ^A^*p* < 0.05 vs. the control group; ^B^*p* < 0.05 vs. the AlCl_3_-intoxicated group, using Tukey's post hoc test.

Real-time PCR results revealed that AlCl_3_ significantly (*p* < 0.05) downregulated the mRNA expression of the *Nrf2* gene in the hepatic and renal tissues as compared with the CON group. In contrast, the administration of MEL 30 minutes before AlCl_3_ led to a significant (*p* < 0.05) upregulation of *Nrf2* mRNA expression in the hepatic and renal tissues as compared with the AlCl_3_ group (Figure 4). The administration of MEL alone improved the *Nrf2* mRNA expression in the hepatic and renal tissues as compared with the CON group.

Next, we examined the nuclear protein expression of Nrf2. As shown in Figure 4, AlCl_3_ significantly decreased the protein expression level of Nrf2. Interestingly, MEL showed a protective effect on the nuclear protein expression level of Nrf2, which was consistent with its mRNA expression patterns. MEL treatment alone significantly increased the expression of Nrf2. Our results demonstrated that MEL increased the protein expression level of Nrf2, particularly under oxidative stress.

### 3.5. Results of Tissue Proinflammatory Cytokines

**Figure 5:**
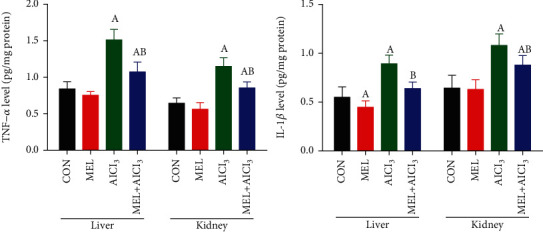
Protective effects of melatonin on aluminum chloride (AlCl_3_-) induced inflammation in the liver and kidney tissues of rats. Data are expressed as mean ± SD values (*n* = 7). ^a^*p* < 0.05 vs. the control group; ^b^*p* < 0.05 vs. the AlCl_3_-intoxicated group, using Tukey's post hoc test.

Figure 5 shows significant (*p* < 0.05) increases in the level of TNF-*α* and IL-1*β* levels in the hepatic and renal tissues of the AlCl_3_ group as compared with the CON group. Pretreatment with MEL significantly (*p* < 0.05) diminished the increases in the levels of TNF-*α* and IL-1*β* as compared with the AlCl_3_-intoxicated rats.

### 3.6. Results of Histopathological Studies

**Figure 6:**
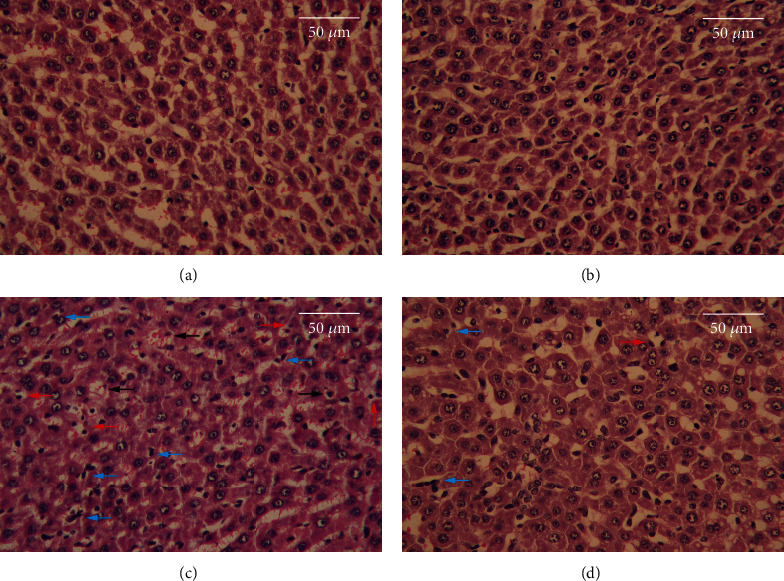
Effects of melatonin on pathological changes in liver tissues of rats exposed to aluminum chloride (AlCl_3_): (a) control, (b) MEL, (c) AlCl_3_, and (d) MEL+AlCl_3_. Magnification at 400x. The red arrow indicated apoptotic hepatocytes, the blue arrow indicates inflammatory cells infiltrated, and the black arrow indicates degenerated hepatocytes.

There were no histological changes detected in the livers of rats in the CON and MEL groups as shown in Figures 6(a) and 6(b), respectively. In contrast, after 8 weeks, the livers of AlCl_3_-treated rats showed severe hepatic necrosis and disarrangement of hepatic lobules. The portal regions also showed massive granular and vesicular degeneration, inflammatory cell infiltration, and vacuolation (Figure 6(c)). MEL pretreatment largely prevented AlCl_3_-induced histopathological alterations in the liver, as evidenced by a reduction in the infiltration of inflammatory cells and hepatocytic injuries (Figure 6(d)).

**Figure 7:**
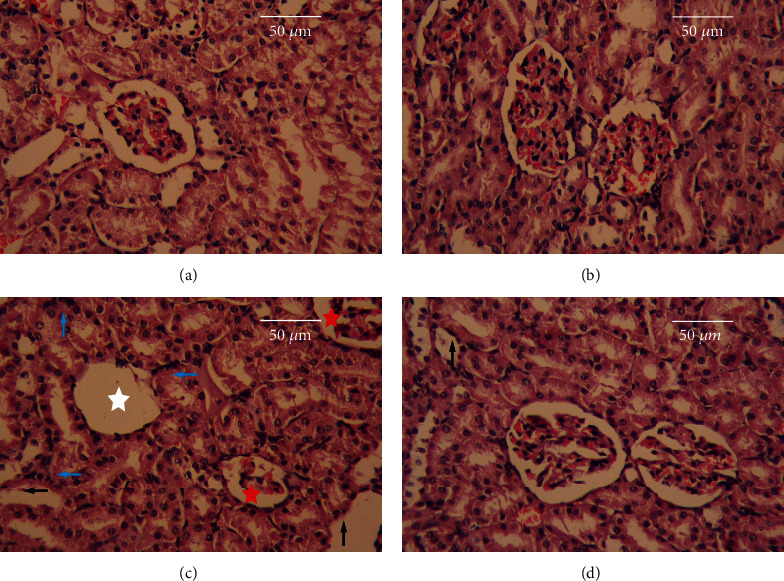
Effects of melatonin on pathological changes in the kidney tissues of rats exposed to aluminum chloride (AlCl_3_): (a) control, (b) MEL, (c) AlCl_3_, and (d) MEL+AlCl_3_. Magnification at 400x. The red star indicates congested glomerulus, the white star indicates degenerated glomerulus, the blue arrow indicates inflammatory cells infiltrated, and the black arrow indicates degenerated renal tubules.

As shown in Figure 7(a), the renal tissues of CON rats had intact renal parenchyma with well-defined glomerular tufts and tubules. AlCl_3_ intoxication induced degeneration and collapse of the glomeruli of the kidney (Figure 7(c)). Pretreatment with MEL mitigated renal histoarchitecture alteration and resulted in a well-formed glomerulus in Bowman's capsule (Figure 7(d)). Moreover, the administration of MEL alone did not cause any histological abnormalities in the kidneys of rats, as seen in Figure 7(b).

### 3.7. Results of Immunohistochemical Studies

**Figure 8:**
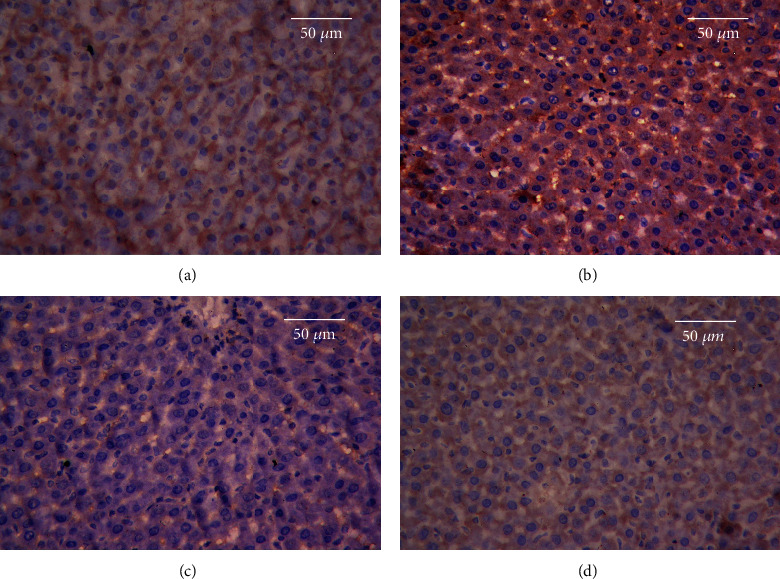
Effects of MEL on Bcl-2 immunostaining of the liver tissues of rats exposed to aluminum chloride (AlCl_3_): (a) control, (b) MEL, (c) AlCl_3_, and (d) MEL+AlCl_3_. Magnification at 400x.

**Figure 9:**
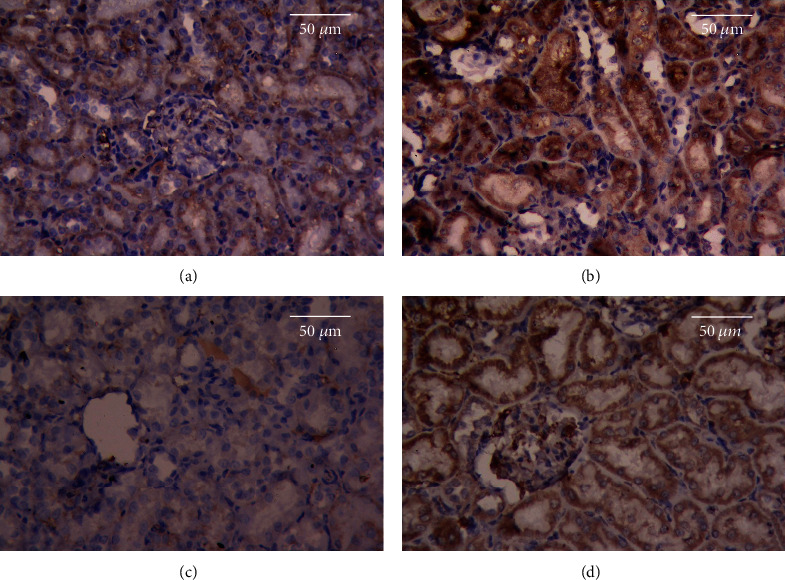
Effects of MEL on the Bcl-2 immunostaining of the kidney tissues of rats exposed to aluminum chloride (AlCl_3_): (a) control, (b) MEL, (c) AlCl_3_, and (d) MEL+AlCl_3_. Magnification at 400x.

Immunohistochemical analysis of Bcl-2 revealed minimal immunoreactivity and normal cell life cycles in the hepatic and renal tissues of the CON group (Figures 8(a) and 9(a), respectively). In contrast, the immunostaining activity for Bcl-2 was significantly diminished in the AlCl_3_ group, which manifested the apoptotic action of Al in the hepatic and renal tissues (Figures 8(c) and 9(c), respectively). However, the immunostaining level of Bcl-2 increased markedly in the rats pretreated with MEL, suggesting that MEL has antiapoptotic properties (Figures 8(d) and 9(d), respectively). Moreover, the livers and kidneys of rats treated with MEL alone showed a markedly higher level of Bcl-2 immunostaining than those of rats in the CON group (Figures 8(b) and 9(b), respectively).

## 4. Discussion

Exposure to Al is extensive owing to the long history of use of this heavy metal in medicine, manufacturing, farming, and water treatment. Al is absorbed by several routes (oral, intranasal, transdermal, and parenteral) [23]. However, little is known about the hepatotoxic and nephrotoxic actions of Al in animals and humans. The current investigation was designed to determine whether MEL could protect the hepatic and renal tissues from Al-induced toxicity.

Our findings revealed that intoxication of rats with AlCl_3_ (34 mg/kg bwt) for eight consecutive weeks resulted in its accumulation in the hepatic and renal tissues. These findings align with those of Al Kahtani [24], who demonstrated that Al accumulates in the liver and kidney tissues of rats exposed to a high dose of Al.

The present study showed that all rats intoxicated with AlCl_3_ presented a typical pattern of hepatotoxicity, as confirmed by increases in the levels of ALT, AST, and bilirubin in serum, and the elevated serum creatinine and urea levels indicated nephrotoxicity. Abdel Moneim et al. [3] found that the accumulation of Al in organs results in molecular impairment or dysfunction. Our results agree with those of Al-Kahtani and Morsy [25], who reported that the main symptoms of Al hepatotoxicity and nephrotoxicity are elevated levels of liver and kidney function markers. As confirmed by Abdel Moneim et al. [3], liver enzymes are important biomarkers of hepatocellular injury. Yousef et al. [5] concluded that exposure to AlCl_3_ causes liver necrosis, severe destruction to the cellular membranes, and subsequent discharge of intracellular enzymes and bilirubin into the blood circulation. In the present study, AlCl_3_-induced liver and kidney injuries were confirmed by a histopathological assessment, which revealed many deleterious morphological alterations in the liver and kidney tissues of the AlCl_3_ group. These observations were consistent with the findings of Yousef et al. [5] and Morsy et al. [26].

The mechanism of Al toxicity is far from being fully understood, although it has been suggested that ROS is the key player in Al-induced hepatorenal toxicity [25]. Increased ROS levels are attributed to mitochondrial hyperactivity, electron leakage, and enhanced electron transport chain activity. Indeed, ROS attacks almost all cellular components, including membrane phospholipids, and causes LPO [27]. As another possible mechanism, Pérez et al. [28] hypothesized that the toxic effects of Al are mediated by damage to cell membranes. The intracellular increase in ferrous iron levels leads to LPO. Excessive LPO impairs cellular membrane permeability, fluidity, and integrity, which eventually leads to apoptosis.

When free radicals are overproduced, the body defends itself from these radicals by synthesizing enzymatic endogenous antioxidants (such as CAT, SOD, GR, and GPx) or nonenzymatic ones (such as GSH), which represent the first line of defense against free radical damage [29]. In the present study, Al-intoxicated animals demonstrated many markers of oxidative stress as evidenced by increases in the levels of LPO and NO with a simultaneous decrease in the GSH content as well as inhibition in the activities of GPx, SOD, CAT, and GR in the hepatic and renal tissues. This emphasizes the role of free radicals in oxidative cellular damage caused by Al toxicity. These findings are consistent with those of previous studies, which showed that Al intake causes oxidative stress and leads to a decrease in the GSH content and the inhibition of antioxidant enzyme activity in different tissues [5, 25]. This inhibition in the activities of antioxidant enzymes observed in this study may be attributed to the reduced biosynthesis of these enzymes owing to higher intracellular levels of Al and/or the overproduction of free radicals [30].

MEL is the most studied molecule with a well-known antioxidant effect. It may offer protection to the body by quenching free radicals and promoting the synthesis of other endogenous antioxidants. In fact, one molecule of MEL can deactivate two hydroxyl radicals. MEL-derived products are believed to augment MEL in protecting against free radicals [31]. Our findings demonstrate that pretreatment of rats with MEL (5 mg/kg/day) for eight weeks attenuated AlCl_3_-induced hepatic and renal toxicity, as verified by the prevention of Al accumulation in the liver and kidney tissues. Our results are in agreement with those of Romero et al. [32], who postulated that MEL has a chelating effect that may contribute to reducing metal-induced toxicity.

In the present study, pretreatment with MEL considerably inhibited Al-induced liver injury in the rats, as evidenced by reductions in aminotransferase activity and the level of bilirubin. MEL also restored the structures of the hepatic cells and lobules to nearly normal. Furthermore, it significantly decreased the elevation in levels of serum creatinine and urea, leading to a marked improvement in the Al-induced nephrotoxicity and degenerative changes in renal tubules and corpuscles. These findings are in agreement with previous studies, which reported that MEL injection during xenobiotic exposure conferred resistance to the xenobiotic-induced unfavorable effects on the hepatic and renal tissues of rats [33–35].

Our results indicate that MEL treatment restored the balance between oxidant and antioxidant molecules, as reflected by the reduction in LPO and NO levels and the increased activity of antioxidant enzymes and GSH content in the liver and kidney tissues. Our results strongly indicated that MEL can alleviate AlCl_3_-induced oxidative stress by promoting antioxidant enzyme activities and quenching ROS. These findings agree with those of Ko et al. [33] and Fernandez-Gil et al. [36], who demonstrated that MEL not only quenches free radicals but also promotes the activity of antioxidant enzymes, preventing oxidative stress. Tan et al. [37] suggested that the antioxidant effects of MEL result primarily from electron donation and its ability to easily cross morphophysiological barriers. In addition, it can easily reach the different cellular components that enhance the ROS removal effect.

In this study, it was observed that AlCl_3_ intoxication significantly downregulated the expression level of the *Nrf2* gene in the hepatic and renal tissues as compared with the CON group. These data are in agreement with those of Yu et al. [38] and Xu et al. [39], who found that Al toxicity is associated with *Nrf2* deregulation. One of our most interesting results is that the administration of MEL 30 minutes before AlCl_3_ upregulated the expression level of the *Nrf2* gene in the liver and kidney of rats by more than 100% as compared with the AlCl_3_ group. This could suggest that activation of Nrf2 is one of the MEL's key protective mechanisms to relieve AlCl_3_-induced toxicity. Moreover, the effect of MEL on Nrf2 expression in the liver and kidney of the treated rats aligned with the activities of antioxidant enzymes in this study. Our findings agree with those of Yu et al. [38], who suggested that MEL promotes the mRNA expressions of *Nrf2* and its target genes in spleens exposed to AlCl_3_, suggesting that MEL could facilitate the nuclear translocation of Nrf2, which in turn enhances the spleen's antioxidant capacity.

The observed morphological lesions in the hepatic and renal tissues of AlCl_3_-intoxicated rats may explain the increased levels of the proinflammatory cytokines TNF-*α* and IL-1*β* as compared with the CON group. The data of the present study agree with the results of Zhang et al. [40], Justin-Thenmozhi et al. [41], and Hosny et al. [42], who found that Al exposure increased the levels of TNF-*α* and IL-1*β* in the liver and kidney tissues of rats.

It was previously stated that the pathological bases of Al toxicity are oxidative stress and apoptosis [43]. Therefore, we explored the effects of MEL on the expression of the Bcl-2 protein, which plays a crucial role in the regulation of apoptotic cell death. Specifically, Bcl-2 inhibits apoptosis in various cell systems and suppresses caspase activity by either preventing cytochrome C discharges from the mitochondrial matrix and/or binding to the apoptosis-activating factor (APAF-1). Bcl-2 may also attenuate inflammation by impairing the nucleotide-binding domain, leucine-rich repeat protein (NLRP1-) inflammasome activation. Hence, MEL prevents caspase 1 activation and IL-1*β* release [44]. In this study, Bcl-2 protein expression was markedly downregulated in rats exposed to AlCl_3_. Thus, AlCl3 may cause hepatic and renal apoptosis by reducing Bcl-2 expression. Our results are in agreement with those of Li et al. [45].

In the current study, MEL treatment significantly enhanced Bcl-2 expression and thus limited apoptosis and the expression of proinflammatory cytokines such as TNF-*α* and IL-1*β*. Our data suggest that MEL prevented or reversed AlCl_3_-induced apoptosis and inflammation accompanied by oxidative stress, which may be the basis of MEL's protective role against Al-induced pathological alterations to the liver and kidney tissues of rats. Our findings are consistent with those of Xu et al. [46], who demonstrated that MEL suppresses apoptosis and oxidative stress through a SIRT1-dependent mechanism in mouse Leydig cells. Additionally, our findings align with those of Radogna et al. [47], who stated that MEL inhibits apoptosis by regulating apoptosis-related genes and stabilizing matrix metalloproteinases (MMPs), and those of Dutta et al. [11], who reported that MEL attenuated arsenic-induced nephropathy in mice by preventing oxidative stress and inflammatory signaling cascades.

Based on the results of the current study, we can conclusively state that MEL has the potential to prevent Al-induced hepatotoxicity and nephrotoxicity. This effective role of MEL is related to its free radical scavenging activity, stimulation of Nrf2 and antioxidant enzymes, and inhibition of apoptosis, inflammation, and accumulation of Al ions. Thus, MEL is of critical importance for the development of novel therapeutic strategies targeting the toxicity associated with chronic Al exposure.

## Figures and Tables

**Table 1:** Details of primer sequences analyzed in real-time PCR.

Name	Sense (5′---3′)	Antisense (5′---3′)
*β*-Actin	GCAGGAGTACGATGAGTCCG	ACGCAGCTCAGTAACAGTCC
Nrf2	TTGTAGATGACCATGAGTCGC	ACTTCCAGGGGCACTGTCTA

**Table 2:** Protective effects of melatonin on aluminum chloride (AlCl_3_-) induced alternation in the liver and kidney markers in serum of rats.

Parameter	CON	MEL	AlCl_3_	MEL+AlCl_3_
ALT (U/l)	59.31 ± 3.1	57.56 ± 4.93	87.65 ± 8.75^a^	61.87 ± .5.51^b^
AST (U/l)	47.78 ± 2.77	45.76 ± 3.65	76.54 ± 6.57^a^	55.36 ± 3.63^b^
Bilirubin (mg%)	0.98 ± 0.05	0.87 ± 0.07	1.9 ± 0.75^a^	1.1 ± 0.64^b^
Urea (mg%)	43.81 ± 8.54	45.58 ± 7.9	76.53 ± 9.65^a^	57.42 ± 7.58^ab^
Creatinine (mg%)	0.55 ± 0.09	0.61 ± 0.03	3.2 ± 0.73^a^	1.46 ± 0.46^ab^

Data are expressed as mean ± SD values (*n* = 7). ^a^*p* < 0.05 vs. the control group; ^b^*p* < 0.05 vs. the AlCl_3_-intoxicated group, using Tukey's post hoc test.

## Data Availability

All relevant data are within the paper.
