# Correction to “The Protective Effects of Melatonin on Aluminum‐Induced Hepatotoxicity and Nephrotoxicity in Rats”

**DOI:** 10.1155/omcl/9784046

**Published:** 2026-06-23

M. S. Othman, M. A. Fareid, R. S. Abdel Hameed, and A. E. Abdel Moneim, “The Protective Effects of Melatonin on Aluminum‐Induced Hepatotoxicity and Nephrotoxicity in Rats,” *Oxidative Medicine and Cellular Longevity* 2020, no. 1 (2020): 1–12, https://doi.org/10.1155/2020/7375136.

In the Discussion section, Figure 9d was incorrect. During the preparation of the manuscript, Figure 9d was mistakenly duplicated from an image published in an earlier study by the same author group [1]. The correct Figure 9d is shown below:

**Figure:**
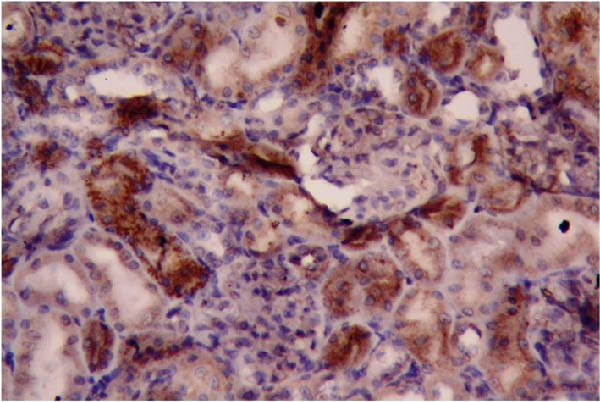


We apologize for this error.
